# The Use of Adjunct Therapies with Surgery for Gastroenteropancreatic Neuroendocrine Tumors: Indications, Timing, and Outcomes

**DOI:** 10.3390/biomedicines14092105

**Published:** 2026-09-18

**Authors:** Kathryn Cavallo, Naris Nilubol

**Affiliations:** 1 Surgical Oncology Program, National Cancer Institute, National Institutes of Health, Bethesda, MD 20892, USA; 2 Endocrine Oncology Branch, Center for Cancer Research, National Cancer Institute, National Institutes of Health, Bethesda, MD 20892, USA

**Keywords:** GEP-NET, pancreatic NET, small bowel NET, PRRT, cytoreduction, somatostatin analogues, metastatic NET, neoadjuvant therapy, adjuvant therapy

## Abstract

**Background/Objectives:** Gastroenteropancreatic neuroendocrine tumors (GEP-NETs) are rare biologically heterogeneous neoplasms but with increasing incidence in recent years. Surgery remains the backbone of treatment; however, long-term recurrence is common even after curative resection. Despite expanding treatment options, the optimal perioperative integration of adjunct therapies in conjunction with surgery remains incompletely defined, as available evidence is largely retrospective and heterogeneous. This review critically synthesizes available evidence on the role of perioperative therapies in the surgical management of GEP-NENs, identifies key knowledge gaps, and highlights areas where multidisciplinary decision-making is required. **Methods:** A targeted narrative literature review was performed in PubMed/MEDLINE (2001–2026), supplemented by ClinicalTrials.gov and major society guidelines. Predefined search terms were used to include “gastroenteropancreatic neuroendocrine tumor”, “GEP-NET”, “pancreatic neuroendocrine tumor”, “surgery”, “palliative surgery”, “neoadjuvant”, “adjuvant”, “PRRT”, “somatostatin analogue”, “chemotherapy”, “targeted therapy”, “immune checkpoint inhibitor”, and “liver-directed therapy”. Studies were selected based on relevance, with emphasis on comprehensive reviews, key clinical studies, and major guideline statements. This approach was chosen due to varied literature and scarce high-quality prospective evidence. **Results:** Neoadjuvant PRRT and CAPTEM-based chemotherapy demonstrate encouraging activity in selected patients with SSTR-positive or borderline resectable pNETs, though evidence is predominantly retrospective and R0 resection rates are inconsistent across modalities. Adjuvant systemic therapy lacks high-quality evidence of benefit in well-differentiated GEP-NETs whereas platinum-based chemotherapy following resection of NEC represents the only setting where adjuvant treatment is commonly recommended, albeit without randomized trial support. Liver-directed therapies, including thermal ablation, TACE, and TARE, play complementary roles in selected patients with hepatic metastases. The overall evidence base is largely retrospective and heterogenous. Management should be individualized through multidisciplinary discussion.

## 1. Introduction

Gastroenteropancreatic neuroendocrine neoplasms (GEP-NENs) encompass a biologically diverse group of malignancies whose incidence has risen steadily over recent decades. Although complete surgical resection remains the cornerstone of treatment, long-term follow-up studies demonstrate substantial locoregional and distant recurrence rates, even after definitive surgery. Additionally, a significant subset of patients present with metastatic disease in which operative management may be pursued for symptom control, prevention of complications, and selected cytoreductive intent. In recent decades, systemic treatments for GEP-NENs have expanded to include somatostatin analogues, cytotoxic chemotherapy, targeted therapy using small-molecule inhibitors, and peptide receptor radionuclide therapy (PRRT). While these advances have improved survival and quality of life for many patients, their use in conjunction with surgery remains unclear. This review summarizes the current evidence supporting adjunct therapies with surgery for GEP-NENs, with emphasis on the indications for neoadjuvant and adjuvant approaches, integration with cytoreductive strategies in metastatic disease, perioperative safety considerations, and ongoing clinical trials.

## 2. Methods

A targeted narrative literature search was performed in PubMed/MEDLINE from 2001–2026, with supplemental searches of ClinicalTrials.gov and major society guidelines. The following search terms were applied to titles and abstracts: “gastroenteropancreatic neuroendocrine tumor”, “GEP-NET”, “pancreatic neuroendocrine tumor”, “surgery”, “palliative surgery”, “neoadjuvant”, “adjuvant”, “PRRT”, “somatostatin analogue”, “chemotherapy”, “targeted therapy”, “immune checkpoint inhibitor”, and “liver-directed therapy”. The search covered publications from January 2001 to July 2026. No language restriction was applied, but only English-language manuscripts were ultimately reviewed. Eligible study designs included randomized controlled trials (RCTs), prospective or retrospective cohort studies, case–control studies, and meta-analysis reporting data specific to the perioperative setting. Bibliographies of retrieved articles and relevant international guidelines (ENETS, NANETS, NCCN, ESMO) were hand-searched to identify additional eligible studies. Studies were included if they reported outcomes in perioperative context defined as data specifically pertaining to the neoadjuvant, adjuvant, or intraoperative setting. Studies reporting exclusively on patients with advanced, inoperable, or metastatic disease without a surgical component were excluded from perioperative subsections but are referenced in the general overview subsections to provide mechanistic and efficacy context. Retrospective studies with fewer than 10 patients in the perioperative cohort were also excluded to minimize reporting bias. This review is narrative in design. Although a systematic search strategy was employed to maximize retrieval of literature, no formal screening flowchart was produced. The synthesis reflects the authors’ critical appraisal rather than systematic protocol. Evidence from advanced disease and landmark trials (i.e., NETTER, PROMID, CLARINET) is presented in the general overview subsections to establish biological plausibility and treatment context but are clearly distinguished from evidence derived specifically from the perioperative period.

## 3. Definition and Epidemiology

Neuroendocrine tumors (NETs) are heterogeneous tumors that arise from secretory cells of the neuroendocrine system. These include well-differentiated neuroendocrine tumors (NETs, grades G1–G3) and poorly differentiated neuroendocrine carcinomas (NECs), which are biologically and clinically distinct entities [1]. For clarity, the term GEP-NET is used throughout this review to refer to well-differentiated tumors, while GEP-NEC refers specifically to poorly differentiated carcinomas. The broader term GEP-NEN is used when both groups are addressed together. GEP-NETs are neuroendocrine tumors that arise from the gastrointestinal tract and pancreas, specifically [2]. GEP-NETs may be functional or non-functional, and are most frequently found in the small intestine (30.8%), followed by the rectum (26.3%), colon (17.6%), pancreas (12.1%), and finally the appendix (5.7%) [3]. With diagnostic advancements, the age-adjusted annual incidence of GEP-NETs has increased from 1.09 to 6.98 per 100,000 persons in the last 40 years, partly due to a widespread use of imaging studies, underscoring the need for continued research and optimized treatment strategies [4].

## 4. Role of Surgery

### 4.1. Surgery for Radical Intent

Tumor grade and differentiation are key determinants of prognosis and treatment selection in GEP-NENs. Early-stage disease is generally associated with favorable outcomes, whereas survival in advanced disease is more dependent on tumor biology, primary site, and resectability [2]. For most patients with well-differentiated G1–G2 tumors and selected patients with G3 disease, surgery remains the cornerstone of treatment as it can result in long-term survival and symptom control in functional tumors [2,5,6]. In a large retrospective cohort of 936 patients undergoing a first curative-intent resection of a GEP-NET, overall survival was 92.2% at 3 years, 85.3% at 5 years, and 68.1% at 10 years; however, recurrence remains common, occurring in up to 48.5% at 10 years [7]. For localized disease, surgical principles vary by primary site but consistently aim to achieve complete (R0) resection. In small bowel NETs, segmental resection with en bloc mesenteric lymphadenectomy and careful intraoperative evaluation for multifocal disease is critical [8,9]. In pancreatic NETs (pNETs), resection or enucleation is generally recommended when tumors are functioning and/or ≥2 cm. Thus, active surveillance is appropriate for small (<2 cm), pancreatic NETs [10,11]. For gastric and colorectal NETs, management is more size- and risk-dependent, with smaller, low-grade lesions often amenable to endoscopic resection and larger or higher-risk tumors requiring anatomical resection with lymphadenectomy [12,13]. Importantly, while R0 resection remains the goal, margin status appears to have a greater impact on recurrence than overall survival, and re-resection to achieve negative margins has not been shown to improve outcomes, supporting the use of parenchyma- or organ-preserving approaches when feasible [14].

While surgery is recommended for patients with resectable disease, additional factors to consider prior to operative intervention include those with functional NETs that require symptom control and in selected patients with metastatic disease. Tumor biology and grade are some of the most important considerations. For example, some societies, such as the North American Neuroendocrine Tumor Society (NANETS), caution against routine surgical resection for poorly differentiated tumors such as neuroendocrine carcinoma (NEC) due to a lack of convincing data demonstrating a survival benefit [6,15,16,17]. While resection may be needed for symptom control, patients should be optimized prior to proceeding with resection, particularly those with carcinoid syndrome, given the increased perioperative risk [13,18]. Finally, patient comorbidities and overall expected life expectancy, as well as any underlying hereditary syndrome, should also be considered. In hereditary syndromes, it is particularly essential to consider the likelihood of recurrence and the need for possible repeated organ resection. In these cases, a parenchymal-sparing resection should be employed when feasible, and observation over resection may even be advisable [5,9,12]. Furthermore, patients with hereditary syndromes may harbor other associated malignancies that require surgical management, which can further influence the timing and sequencing of operative interventions.

It should be noted that the role of surgery in G3 and NEC is controversial with little consensus between society guidelines [19]. Presently, the European Neuroendocrine Tumor Society (ENETS), the National Comprehensive Cancer Network (NCCN), and the European Society of Medical Oncology (ESMO) guidelines recommend consideration for surgical resection in GEP-NET G3 and GEP-NEC when feasible, whereas NANETS does not recommend upfront surgery [6,10,13,20]. While there is consensus among societies that surgery should be avoided in patients with metastatic or locally advanced NECs, the role of surgery for resectable NECs is controversial and should be used selectively after a discussion in a multidisciplinary conference [6,,13,20,21].

To help guide surgical management and surveillance following resection, several groups have developed recurrence-risk models for GEP-NETs. In one multicenter study of 1006 pNETs, symptoms, tumor size, Ki-67, and nodal status were used to stratify patients into low-, intermediate-, and high-risk recurrence groups with distinct recommended surveillance intervals for each group [22]. Similarly, a retrospective review of patients with localized pNETs found that lymph node involvement, histologic grade, tumor size, and Ki-67 were independent predictors of post-resection metastatic recurrence [23]. A second retrospective study of 441 GEP-NETs developed a prognostic score to determine recurrence risk in patients following curative-intent resection [24]. This score included TNM stage, lymph node ratio, margin status, and found significant differences in recurrence-free survival (RFS) between low-, medium-, and high-risk patients, demonstrating the importance of a patient-specific surveillance strategy following surgical resection [24]. Additional markers, including the neutrophil-to-lymphocyte ratio (NLR), have also been found in the preoperative setting to correlate with tumor stage, lymph node metastasis, and tumor grade, offering complementary tools for risk stratification prior to resection [25].

Surgery for GEP-NETs is generally safe with very low reported rates of perioperative mortality [26,27]. The morbidity associated with resection of GEP-NETs, however, is non-negligible, organ-specific, and should be considered along with other patient risk factors in determining appropriateness for resection. Small bowel NET resections have associated morbidity in approximately 13% of cases, with the most common complications including intraabdominal bleeding and ileus [28]. Pancreatic resections or enucleations, however, have a much higher associated morbidity. A systematic review of the literature reported postoperative morbidity in approximately 50% of pancreatic enucleations for all indications, the most common complication being a postoperative pancreatic fistula (POPF) [29]. When looking at all pancreatic enucleations, the POPF rate was higher in patients with pNETs compared to enucleation for other etiologies, emphasizing the particular risk in this patient population [30]. Despite the morbidity risk associated with parenchymal-sparing procedures, complication rates are higher still in patients undergoing pancreatic resection for pNETs, with reported POPF rates of up to 58% [27]. Other common complications following pancreatic resection for pNETs include delayed gastric emptying and postoperative hemorrhage [27].

### 4.2. Metastatic Disease and Palliation

While approximately 50% of patients with GEP-NENs initially present with localized disease, there is no definitive data on the percentage of patients with GEP-NENs initially presenting with resectable disease [4,31]. Up to 27% of patients present with distant metastases at the time of diagnosis, underscoring the importance of considering surgical management, including metastasectomy, in select individuals [4]. Liver is the most common site of GEP-NEN metastases, followed by regional lymph nodes and less common bone, lungs, and peritoneum [32,33]. Liver metastases are associated with worse constitutional symptoms and overall survival [34,35]. As such, patients with metastatic disease may be eligible for surgical interventions to control symptoms or prolong survival. In a retrospective review of the Surveillance, Epidemiology, and End Results (SEER) database, primary resection with metastasectomy was associated with longer disease-specific survival compared to no surgery [36]. Furthermore, metastasectomy has been associated with prolonged survival in patients with recurrent liver metastases [35]. However, these retrospective analyses are inevitably confounded by selection bias.

While resection of the primary tumor in patients with metastatic GEP-NETs may be controversial, it is recommended for symptomatic management when feasible [9,37,38]. Additionally, primary small bowel NETs are typically recommended for resection due to the risk of obstruction even in the presence of distant metastasis [9]. Palliative surgery may also be required to either treat or prevent bowel obstructions or manage symptoms related to carcinoid syndrome. However, it remains debatable whether such prophylactic surgery is necessary to prevent complications. A retrospective review of patients who underwent surgery for small bowel NETs found that emergency resections were required in approximately 8% of cases for obstruction or perforation [39]. A European cohort study of asymptomatic patients with stage IV small bowel NETs attempted to answer the question of whether early surgical intervention could prevent complications. The group compared patients who underwent prophylactic resection for small bowel NETs within six months of diagnosis, in addition to oncologic therapy, to those who were managed with oncologic therapy alone or delayed surgery. The authors found that there was no difference in overall survival and, furthermore, that patients in the prophylactic surgery group had more re-operative procedures (14.3%) compared to those in the delayed surgery group (3.3%), suggesting that prophylactic surgery for stage IV small bowel NETs may not be beneficial [40].

### 4.3. Neoadjuvant vs. Adjuvant Integration

Adjunct therapies may complement surgery in selected patients; however, most available evidence is retrospective and heterogeneous, limiting clear conclusions regarding their optimal use. For the purposes of this review, the following definitions are applied consistently through: neoadjuvant therapy refers to systemic or liver-directed treatment administered prior to planned surgical resection with the intent to downstage disease or improve resectability; adjuvant therapy refers to treatment administered after curative-intent resection in patients with no clinical evidence of residual disease, with the goal of reducing recurrence risk; salvage therapy refers to treatment initiation at the time of confirmed disease recurrence or progression following prior resection. These distinctions are particularly important given that evidence quality and clinical applicability differ substantially across these settings. A comprehensive summary of neoadjuvant therapy, adjuvant therapy, and salvage therapy following post-resection recurrence is summarized in Table 1, Table 2 and Table 3 respectively.

The current guidelines (ENETS, NANETS, NCCN, ESMO, ASCO) do not routinely recommend adjuvant therapy after R0 or R1 resection of well-differentiated GEP-NETs. However, this gap in knowledge is currently being studied in clinical trials (NCT05040360) [41,42,43]. In addition, it is not uncommon to start or continue somatostatin analogues (SSAs) in patients with residual tumors. Thus, the use of adjunct therapy in patients with resectable, well-differentiated GEP-NETs should be personalized and discussed in a multidisciplinary tumor board. In poorly differentiated NEC, adjuvant platinum-based chemotherapy is commonly recommended despite the absence of randomized trials [44].

Neoadjuvant therapy may help downstage and improve resectability in locally advanced GEP-NETs, although this effect is not uniformly seen. A systematic review and meta-analysis assessing the role of neoadjuvant therapy (including PRRT, chemotherapy, and sunitinib) in GEP-NETs found that neoadjuvant therapy may be effective and safe in the treatment of GEP-NETs. Authors reported objective response rates (ORR) of 42% [45]. Interestingly, in this same study, the R0 resection rates for patients were similar between those who received neoadjuvant treatment and those who did not (60% vs. 63%), indicating that neoadjuvant therapy was not associated with an increase in R0 resection [45,46].

The goals of postoperative adjuvant therapy include (1) reduction in locoregional and/or systemic disease recurrence following R0 resection in patients who are at risk for microscopic metastatic or residual disease; and (2) increase in progression-free survival in patients who underwent cytoreduction with clinically detectable residual disease. In the absence of level I evidence of clinical benefits with some conflicting evidence, the use of adjuvant therapy in patients with GEP-NETs undergoing surgery should be individualized based on the risk of disease progression. A retrospective study from US-NETSG did not find any differences in OS and RFS in patients with primary GEP-NETs who underwent curative-intent resection between those who received adjuvant cytotoxic chemotherapy or SSAs and those who did not. [47]. Additionally, a retrospective review of the National Cancer Database (NCD) compared the benefit of perioperative systemic therapy to surgery alone in patients undergoing curative-intent resection of localized pNETs, finding no difference in overall survival (OS) [48]. Risk stratification using clinical variables such as tumor grade, tumor burden, and pace of disease progression should therefore guide individualized treatment decisions.While postoperative adjuvant therapy aims to improve disease progression only after surgery, the additional goal of neoadjuvant therapy is to improve resectability in patients with unresectable or borderline resectable disease.

**Figure 1:**
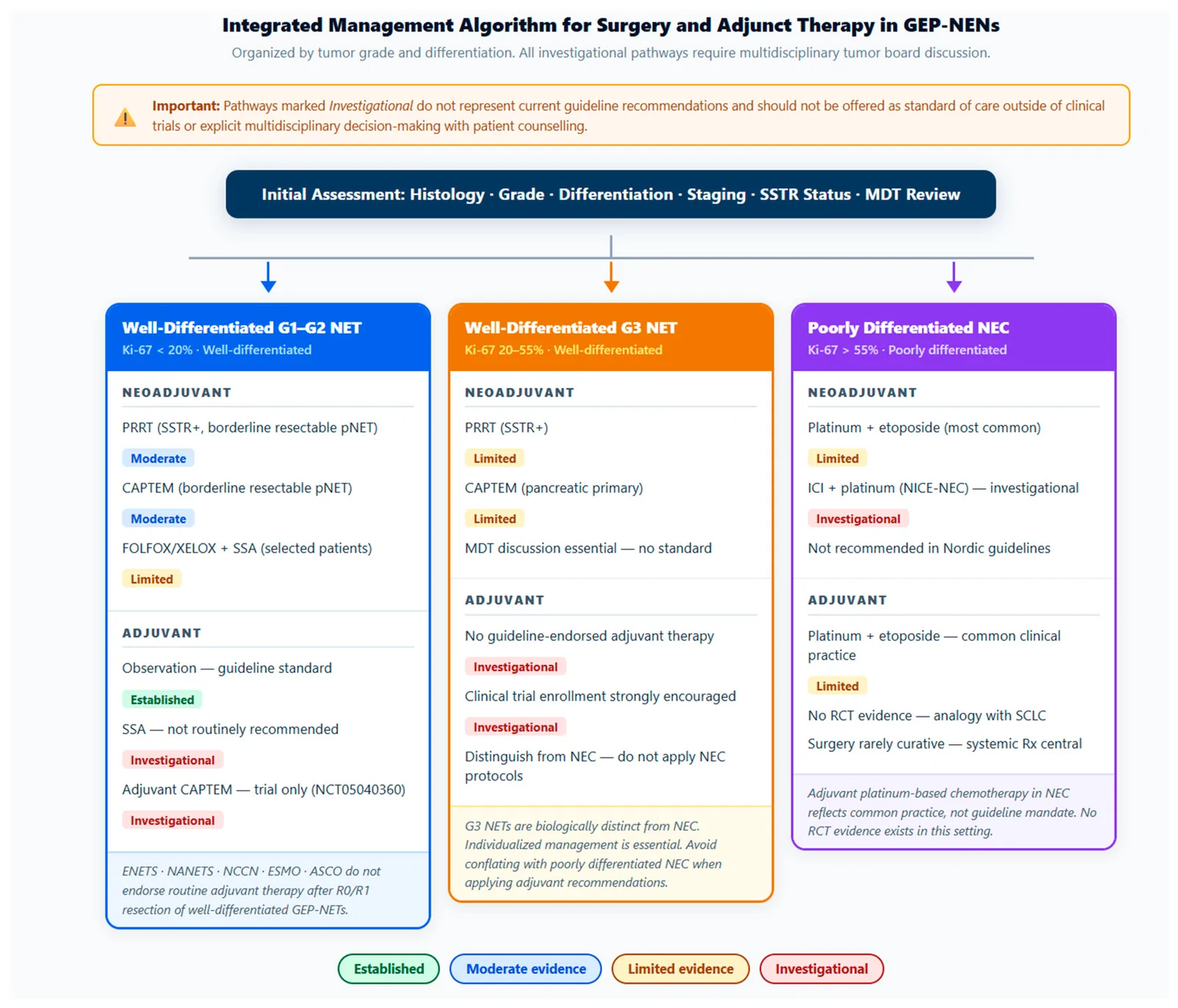
Integrated Management Algorithm for Surgery and Adjunct Therapy in GEP-NETs. Abbreviations: G1, Grade 1; G2, Grade 2; G3, Grade 3; NEC, neuroendocrine carcinoma; SSTR, somatostatin receptor; CAPTEM, capecitabine and temozolomide; PRRT, peptide receptor radionuclide therapy.

Regarding NEC or high-grade GEP-NETs, the data and recommendations for the use of neoadjuvant therapy are not universally accepted. Presently, neoadjuvant therapy is not recommended for the treatment of NEC in the Nordic guidelines, whereas other societies recommend multidisciplinary consideration for its use in poorly differentiated tumors [8,49,50]. A proposed treatment algorithm for the utilization of surgery and ajunct therapy in GEP-NENs is provided in Figure 1.

## 5. Therapies

### 5.1. Chemotherapy

Cytotoxic chemotherapy is used to treat GEP-NETs with rapid disease progression, primarily in the treatment of grade 3 GEP-NETs. The combination of temozolomide and capecitabine (CAPTEM) has demonstrated efficacy in treating GEP-NETs and has shown objective response rates ranging between 30–70% and a significantly longer PFS compared to temozolomide in pNETS specifically [51,52,53,54]. In one retrospective review from Columbia University, Fine et al. report an objective response rate of 61% for patients with metastatic GEP-NETs to the liver [55]. Dacarbazine, when combined with 5-fluorouracil (5-FU), represents an alternative to CAPTEM and has demonstrated similar efficacy in advanced GEP-NETs, with reported objective response rates of approximately 38–39% [56]. Streptozocin-based regimens have also been used in the treatment of pNETs, particularly in combination with other cytotoxic agents or antiangiogenic agents [55,57,58]. Prakash et al. (2017) retrospectively assessed response rates in patients with localized pNETs who received neoadjuvant 5-FU, doxorubicin, and streptozotocin. The authors found that 90% of patients had stable disease (SD) compared to 7% who had a partial response (PR) and 3% who had progression of disease (PD) as defined by the Response Evaluation Criteria in Solid Tumors (RECIST) [59]. FOLFOX has also demonstrated some efficacy in the treatment of advanced GEP-NETs, with reported rates of disease control in 75% of all cases and 91.3% when used as a first-line treatment according to one retrospective series [60]. Additionally, FOLFOX has also demonstrated efficacy in well-differentiated metastatic pNETS that have progressed on prior therapy, with reported overall response rates of 45.2% [61].

Regimens for NEC include platinum-based chemotherapy in addition to etoposide [62,63]. Additionally, folinic acid, fluorouracil, and irinotecan (FOLFIRI) and folinic acid, fluorouracil, irinotecan, and oxaliplatin (FOLFIRINOX) have demonstrated some marginal benefit for patients with metastatic or localized NEC who have progressed on platinum regimens [64,65].

#### 5.1.1. Neoadjuvant Chemotherapy

Chemotherapy has also been used in the neoadjuvant setting. A single-center retrospective review in patients with pNETs and borderline resectable disease demonstrated successful surgical resection following neoadjuvant CAPTEM with or without radiation [66]. CAPTEM was further validated in another retrospective review in which patients with locally advanced or resectable metastatic pNETs were treated with neoadjuvant CAPTEM. The authors found that 43% of patients exhibited a partial radiographic response and 87% underwent resection. They reported a median PFS of 28.2 months and 5-year OS of 63%, suggesting that CAPTEM may be useful in the neoadjuvant setting for patients with borderline resectable or metastatic pNETs [54,55,67]. The combination of chemotherapy with other modalities has also demonstrated some efficacy in the neoadjuvant setting. A retrospective review of patients with GEP-NETs initially treated with combination oxaliplatin-fluoropyrimidine chemotherapy and SSA reported a disease control rate of 87.5%, with 28% of patients demonstrating downstaging to radiographic resectability, highlighting the potential utility of chemotherapy in the neoadjuvant setting [68].

#### 5.1.2. Adjuvant Chemotherapy

Regarding standard cytotoxic chemotherapy, adjuvant treatment is not recommended in low- or intermediate-grade GEP-NETs, given low recurrence rates and a lack of data supporting any benefit of postoperative therapy [13,69]. A retrospective review assessed the efficacy of adjuvant streptozotocin and 5-FU following resection of liver metastasis for GEP-NETs found no statistically different significance in RFS or OS between those that received adjuvant chemotherapy and those that did not (RFS at 5 years 20% vs. 38% *p* = 0.36, OS at 5 years 96% v. 76%, *p* = 0.58) [70].

However, adjuvant chemotherapy is commonly recommended following resection in patients with NECs, given the high risk of recurrence and systemic metastasis following resection, despite a lack of randomized controlled trials demonstrating their benefit [6,13,44,69]. Given that surgery is rarely curative for patients with NEC, adjuvant treatment with platinum and etoposide should be considered after surgery due to the high risk of recurrence [71].

Clinical Implication: CAPTEM-based neoadjuvant chemotherapy may be considered in selected patients with borderline resectable or locally advanced pNETs. Adjuvant cytotoxic chemotherapy is not recommended for well-differentiated GEP-NETs but is a standard consideration following resection of NEC. FOLFOX/XELOX remains investigational in the perioperative setting.

### 5.2. Somatostatin Analogues

For patients with advanced or unresectable functional GEP-NETs, SSAs are the standard first-line treatment [6,19]. The PROMID trial was a double-blind, placebo-controlled phase 3 trial in which 85 patients with well-differentiated, advanced midgut NETs received either octreotide long-acting release or placebo. They reported a longer median time to progression in the treatment arm compared to the placebo arm, supporting the use of SSAs in midgut NETs (14.3 months vs. 6 months, *p* < 0.001) [72]. The CLARINET study was another double-blind, placebo-controlled phase 3 trial in which 204 patients with progressive metastatic GEP-NETs with Ki-67 <10% were randomized to receive either lanreotide or placebo. The authors found that lanreotide was associated with longer progression-free survival (PFS) compared to the placebo (median PFS not reached vs. 18 months, *p* = 0.001) [73]. It should be noted that both PROMID and CLARINET enrolled patients with advanced, unresectable disease. Therefore, neither trial addresses specifically the neoadjuvant or adjuvant role of SSAs following surgical resection. The perioperative evidence is discussed separately in subsequent subsections.

#### 5.2.1. Neoadjuvant SSA

Although the PFS was longer in treated groups in the above trials, only 2–5% developed a partial response to SSA treatments. Thus, the use of SSA is mainly as postoperative adjunct therapy, not as neoadjuvant treatment, to control disease progression. The treatment efficacy of SSAs in NEC or G3 NETs, however, remains controversial due to limited data [19,74,75].

#### 5.2.2. Adjuvant SSA

SSA is the most used adjuvant treatment for GEP-NETs; however, data for its use in the adjuvant setting are conflicting. Two notable retrospective studies have evaluated adjuvant SSA therapy in resected GEP-NETs, yielding discordant results. Gao et al. reported a significant improvement in recurrence-free survival with adjuvant long-acting octreotide in 130 patients with resected G2 pancreatic NETs (R0 or R1 resection), with a notably higher proportion of patients with vascular and perineural invasion in the treatment group at baseline [76]. In contrast, Guo et al. found no significant DFS or OS benefit from adjuvant long-acting octreotide in a larger multicenter cohort of non-metastatic G2 pNETs, despite the inclusion of patients with adverse pathological features including lymph node positivity and tumor size > 2 cm [77]. These discrepancies are likely attributable to differences in patient selection—particularly the proportion of patients with lymph node metastases and vascular invasion—as well as differences in SSA duration (6–12 months in Gao et al. vs. variable duration in Guo et al.) and the absence of SSTR expression data in both cohorts. Notably, a subgroup analysis within the Guo et al. cohort suggested that patients with lymph node metastasis or Ki-67 <10% may derive a DFS benefit from adjuvant octreotide, though this finding requires prospective validation [77].

A third retrospective study by Wang et al. identified G3 histology, pancreatic duct dilation, and perineural invasion as independent predictors or early recurrence in resected pNETs and found that adjuvant SSA was associated with a lower recurrence rate in this high-risk subgroup (*p* < 0.001) [78]. Taken together, current evidence does not support routine postoperative SSA therapy following complete resection of GEP-NETs, and no major guideline endorses this practice. The subgroup findings from Guo et al. and Wang et al. identify potential benefit in patients with G2 pancreatic NETs, lymph node involvement, Ki-67 <10%, and high-risk pathological features such as vascular or perineural invasion are strictly hypothesis-generating, and should not be used as established criteria for patient selection outside of a clinical trial context [79]. Prospective randomized trials stratifying patients by tumor grade, primary site, Ki-67 index, lymph node status, and SSTR2 expression are needed before any recommendations can be made.

The timing of when to resume SSAs following surgical resection also remains controversial. A retrospective study looking at the optimal timing of SSA resumption in patients with metastatic GEP-NETs who underwent surgical debulking found that small bowel neuroendocrine tumors had longer PFS in patients who did not immediately resume SSAs compared to those who did (34 vs. 16 months, *p* = 0.02) [80]. While the results of this study may support the delayed resumption of SSAs, it should be noted that the authors did not control for tumor grade or other confounding variables that may have made providers more likely to resume SSAs immediately. The absence of level 1 evidence warrants a prospective randomized, controlled trial.

Clinical Implication: SSAs have no established neoadjuvant role. Adjuvant SSA use is not guideline-endorsed and should be considered investigational. Resumption of SSA following surgical debulking (R1 or R2 resection) to control tumor progression or symptoms related to hormonal excess is common practice but not supported by level I evidence.

### 5.3. Targeted Therapies

Targeted therapies for GEP-NETs primarily include mechanistic target of rapamycin (mTOR) inhibitors and multitargeted tyrosine kinase inhibitors (TKIs) [19]. Everolimus is an oral mTOR inhibitor, which was approved by the FDA to be used in patients with advanced GEP-NETs. RADIANT-2 was the first phase 3 clinical trial in which 429 patients with hormonally active NETs were randomly assigned to receive either everolimus plus octreotide or placebo plus octreotide. While there was no association with OS, median PFS was slightly longer in the treatment arm compared to the placebo arm (PFS 16.4 vs. 11.4 months, HR 0.77, *p* = 0.26) [81,82]. This study was then expanded upon in the RADIANT-3 and RADIANT-4 clinical trials, in which patients with either low or intermediate-grade pNETs or advanced, progressive, well-differentiated, nonfunctioning GE-NETs, respectively, were randomized to everolimus vs. placebo. Both groups demonstrated a longer median PFS in the everolimus group, leading to its FDA approval for NETs in these populations [83,84]. It should be noted, however, that all RADIANT trials enrolled patients with advanced, unresectable disease, and therefore the perioperative use of everolimus has not been prospectively evaluated. Evidence for its neoadjuvant or adjuvant application is limited to case reports described in sections below. TKIs such as sunitinib were also approved for the treatment of advanced, progressive pNETs after demonstrating improvement in PFS. A double-blind, placebo-controlled, phase 3 trial in this patient population demonstrated a significant improvement in median PFS in the treatment group compared to placebo (11.1 vs. 5.5 months, HR 0.42, *p* < 0.001) [85]. The rates of objective response in these trials ranged from 2% to 9.3%. While both mTOR inhibitors and TKIs are used for the management of GEP-NETs, no data supports their use in NEC [6,86,87].

Sequential systemic treatments that combine different regimens have been studied. Capdevila et al. (2025) recently conducted a randomized, open-label study comparing everolimus followed by streptozotocin and 5-FU (STZ/5-FU) or the reverse sequence in pNETs. The preliminary results showed no significant differences in PFS between the two groups. Chemotherapy as the first treatment, however, had a higher ORR compared to everolimus, suggesting that STZ/5-FU may be used as the first option when downstaging [88]. It should be noted, however, that the number of patients who went on to resection following these regimens was not reported, and there is no data on the safety or outcomes of surgical resection following neoadjuvant STZ/5-FU and everolimus.

#### 5.3.1. Neoadjuvant Targeted Therapies

The described use of neoadjuvant mTOR inhibitors and TKIs in GEP-NETs is largely limited to case reports and small retrospective series, with a clear absence of prospective studies in the literature. Neoadjuvant everolimus and sunitinib have been described in cases of locally advanced pNET with vascular involvement, with reports of occasional tumor stabilization allowing for subsequent resection [89,90]. Objective radiographic responses, however, are infrequent given the primarily cytostatic mechanism of these agents.

#### 5.3.2. Adjuvant Targeted Therapies

No prospective randomized data support the use of adjuvant mTOR inhibitors or TKIs following curative-intent resection of GEP-NETs. As previously mentioned, one retrospective analysis found no significant benefit of perioperative systemic therapy, including targeted agents, in patients undergoing surgery for localized pNETS [48]. While adjuvant CAPTEM is currently under investigation for high-risk resected pNETs, no completed trial addresses adjuvant TKIs or mTOR inhibitors specifically [91]. Current guidelines from ENETS, NCCN, ESMO, and NANETS do not recommend adjuvant targeted therapy following R0 or R1 resection of well-differentiated GEP-NETs [6,10,13,20].

Clinical Implication: Neoadjuvant and adjuvant use of mTOR inhibitors and TKIs is not supported by any prospective data and is not recommended outside of clinical trial settings.

### 5.4. Peptide Receptor Radionuclide Therapy

PRRT has been widely used for somatostatin receptor (SSTR) positive metastatic or unresectable well-differentiated GEP-NETs [92]. PRRT is a form of systemic radiotherapy in which radionuclide isotopes are chelated to an SSTR-targeted carrier molecule, thereby allowing for targeted delivery of radiotherapy to tumor cells that express high levels of SSTRs [93]. In the landmark NETTER-1 trial, patients with inoperable, metastatic midgut neuroendocrine tumors were randomized to receive either ^177^Lu-DOTATATE plus octreotide or octreotide alone. In their preliminary analysis, the authors found that the treatment group had higher 20-month PFS rate and ORR compared to the control group (PFS 65.2% vs. 10.8% (95% CI 50–76.8) and ORR 18% vs. 3% (*p* < 0.001), respectively) [92]. The NETTER-2 trial included metastatic or locally advanced GEP-NETs that were histologically proven G2 or G3. The authors found that the treatment group had a longer median PFS compared to the control group (22.8 vs. 8.5 months, *p* < 0.0001), supporting the use of PRRT as first-line therapy for SSTR-positive metastatic or locally advanced GEP-NETs [94]. Several other studies have demonstrated the efficacy of PRRT in treating strongly SSTR-expressing G3 NETs as well as NEC with low Ki-67 index, influencing guideline recommendations [95,96,97,98]. The phase III COMPETE trial (NCT03049189) recently reported its primary results demonstrating that ^177^Lu-edotreotide significantly improved PFS compared to everolimus in patients with inoperable, progressive G1 or G2 SSTR-positive GEP-NETs (HR 0.67; 95% CI 0.48–0.95; *p* = 0.022). The ORR was also significantly higher with ^177^Lu-edotreotide on central review (21.9% vs. 4.2%; *p* < 0.001) with a lower rate of treatment-emergent adverse events with ^177^Lu-edotreotide compared to everolimus (82.5% vs. 97.0%) [99,100]. Of note, COMPETE, NETTER-1, and NETTER-2 trials enrolled patients with inoperable or metastatic GEP-NETs and did not address perioperative use, although the results further support the efficacy and tolerability of PRRT in GEP-NETs and provide important context for its evolving role in the treatment landscape. Dedicated perioperative studies evaluating PRRT in the neoadjuvant and adjuvant settings are discussed in the subsections below.

#### 5.4.1. Neoadjuvant PRRT

In a systematic review, Yan et al. (2024) found that PRRT was among the most effective neoadjuvant treatment modalities in a pooled analysis [45]. Additionally, preliminary results of the NEOPLANET study have found generally good response rates to neoadjuvant PRRT with a safe surgical profile. In this multicenter, phase II study, patients with resectable or potentially resectable pNETs were given neoadjuvant PRRT followed by surgery. Eighteen of 31 (58%) patients demonstrated a partial response, with no patients having a progressive disease. The authors report postoperative complications occurring in 21 out of 29 patients, with severe complications occurring in only seven patients in the absence of any perioperative mortalities [101]. A more recent, larger meta-analysis conducted by Lee and Kim (2025) reported a pooled ORR of 39.1%, disease control rate (DCR) of 89%, and R0 resection rate of 69% for neoadjuvant PRRT [102]. Additionally, Partelli et al. (2018) reported that neoadjuvant PRRT was safe in patients with pNETs undergoing surgery, with similar complication rates (45% vs. 60%, *p* = 0.032). They observed that neoadjuvant PRRT was associated with a lower risk of developing pancreatic fistula (25% vs. 65%, *p* = 0.011) [103]. Collectively, these results support the safety and efficacy of neoadjuvant PRRT for GEP-NETs in selective patient populations.

#### 5.4.2. Adjuvant PRRT

While PRRT has been studied as a neoadjuvant therapy, there is little to no data assessing its utility or efficacy following curative-intent surgery. There is data, however, on the efficacy of PRRT in the salvage setting for recurrence or progression following surgical resection. A recent retrospective study assessed the OS and PFS for patients who received PRRT for progression or recurrence following resection of GEP-NETs. The authors found that PRRT was associated with longer PFS (32.4 vs. 11.0 months, *p* < 0.001) and OS (49.8 vs. 38.4 months, *p* = 0.009) compared to other salvage therapies or no treatment [104]. The efficacy of adjuvant PRRT following surgical debulking for low-grade, metastatic GEP-NETs is currently under investigation (NCT06016855).

Clinical Implication: Neoadjuvant PRRT may be considered in selected patients with SSTR-positive, borderline resectable or locally advanced pNETs at high-volume centers. Adjuvant PRRT following curative resection remains investigational. PRRT has demonstrated benefit in the salvage setting following post-resection recurrence.

### 5.5. Immune Checkpoint Inhibitors

Immune checkpoint inhibitors (ICIs) have demonstrated efficacy across multiple cancers. However, response rates in GEP-NETs remain modest across numerous studies [105,106,107,108]. The phase II KEYNOTE-158 study evaluated pembrolizumab across multiple tumor types, including 107 patients with advanced, well-differentiated NETs. After a median follow-up of 24.2 months, the objective response rate was 3.7%, with all responses classified as partial and no complete responses reported [106]. Thus, ICIs remain investigational in GEP-NETs, with limited activity in unselected advanced disease and no established role as adjuvant or neoadjuvant therapy around curative-intent surgery. It should be noted, however, that there is emerging data on ICIs in conjunction with platinum-based chemotherapy in G3 NET. Preliminary results of the NICE-NEC trial (GETNET1913) evaluated nivolumab combined with cisplatin or carboplatin as first-line therapy in unresectable or metastatic G3 GEP-NETs, and reported an ORR of approximately 54% and median PFS of 5.7 months in the initial analysis [109]. The final OS results have confirmed the efficacy of this combination in this patient population [110]. Whether this treatment regimen has a role in the perioperative management of resectable NEC, however, remains unexplored and warrants prospective evaluation.

Clinical Implication: ICIs have no established role in the perioperative management of GEP-NETs. Their use as neoadjuvant or adjuvant therapy is strictly investigational and should not be offered outside of a clinical trial.

### 5.6. Liver-Directed Therapies

Liver metastases are present in a substantial proportion of patients with GEP-NENs at diagnosis and are associated with worse prognosis and symptom burden [3,111]. Commonly employed therapies include transarterial chemoembolization (TACE), transarterial embolization (TAE), and radioembolization. These modalities may be performed in the pre or postoperative settings, as well as integrated intraoperatively with resection of primary tumor or additional metastatic disease.

For patients with resectable liver metastases, hepatic resection with curative or cytoreductive intent remains the standard surgical approach when anatomically feasible. However, complete R0 hepatic resection is only achievable in approximately 10–25% of patients with GEP-NEN liver metastases [112]. In patients with unresectable disease, cytoreductive hepatectomy remains clinically meaningful, with early series establishing a 90% cytoreduction threshold as the operative benchmark. Subsequent retrospective series have reported a lower threshold of >70% cytoreduction as both achievable and clinically beneficial. Scott et al. evaluated 188 cytoreductive procedures for GEP-NET liver metastases and found that >70% cytoreduction was associated with significantly improved OS compared to <70% cytoreduction (median OS 134 vs. 38 months) [113]. This supports the argument that tumor biology rather than precise cytoreduction percentage may be the primary driver of long-term outcomes. This finding is reflected in NANETS consensus guidelines, which recommend pursuing cytoreduction using parenchymal-sparing techniques when >70% debulking can be achieved. The largest modern series evaluation of cytoreductive hepatectomy for GEP-NET liver metastases is a retrospective analysis of 522 patients at Mayo Clinic by Gudmundsdottir et al., and demonstrated durable symptomatic relief and prolonged survival [114]. Of note, however, the association between cytoreduction extent and OS was nonsignificant on multivariable analysis, while high Ki-67, pancreatic primary, and extrahepatic disease remained independently predictive, reinforcing that patient selection based on tumor biology may be as if not more important than any debulking threshold [114].

#### 5.6.1. Neoadjuvant/Conversion Liver-Directed Therapy

For patients who are not candidates for immediate surgical resection, liver-directed therapies such as image-guided thermal ablation, transarterial chemoembolization (TACE), transarterial embolization (TAE), and transarterial radioembolization (TARE) may be used in a neoadjuvant or adjunctive setting to reduce tumor burden and improve symptom control. Bösch et al. reported a series of eight patients with GEP-NET liver metastases who underwent radioembolization followed by planned hepatic resection and demonstrated a mean OS of 25.1 months after combined therapy [115]. These therapies may facilitate downstaging in selected patients or provide disease control when surgery is not immediately feasible. It should be noted, however, that the NET-Liver-Metastases Consensus Conference reviewed all available peer-reviewed evidence and concluded that TAE, TACE, and TARE are all supported by moderate-quality evidence for disease control and symptomatic benefit in this patient population without any one modality being found superior in terms of imaging response, symptomatic response, or survival impact [116].

#### 5.6.2. Adjuvant Liver-Directed Therapy

As mentioned, liver-directed therapies may be used in the adjuvant setting following resection of primary tumor or metastatic disease; however, evidence addressing specific liver-directed therapies following resection for GEP-NET liver metastases remains limited. In practice, providers may elect to use intra-arterial therapies following cytoreduction in patients where cytoreductive thresholds are not met, with the goal of providing disease control of residual hepatic tumor. The survival benefit of this approach, however, has not been evaluated by any prospective studies.

Repeat hepatic resection for recurrent liver metastases has, however, been well studied. Multiple retrospective series have demonstrated that repeat hepatectomy is technically feasible and associated with outcomes comparable to the index procedure with durable symptomatic relief and prolonged survival reported in select cases [112]. For patients with recurrent liver metastases who are not surgical candidates, ablative or intra-arterial techniques may be used in conjunction with SSA and PRRTs as salvage therapy, although their use should be guided by multidisciplinary discussion. Of note, there are no randomized trials evaluating adjuvant liver-directed therapy following curative or cytoreductive hepatic surgery for GEP-NETs, illustrating a significant unmet evidence gap.

#### 5.6.3. Intraoperative Liver-Directed Therapy

Intraoperative thermal ablation may be used as a complementary, parenchymal-sparing approach that extends the reach of surgical cytoreduction. Radiofrequency ablation (RFA) and microwave ablation (MWA) may be particularly helpful for small (<3–4 cm), centrally located lesions not otherwise amenable to resection. Reported local recurrence rates following MWA of GEP-NET liver metastases are as low as 3–10% [117]. Combined resection and ablation for liver metastases in the GEP-NET patient population has been found to be safe in the perioperative period without significant increase in infection, bleeding, or bile leak rates, supports its use to expand the number of patients eligible for surgical debulking [118].

Clinical Implication: Intraoperative thermal ablation is an established adjunct to hepatic resection for NELM. Neoadjuvant intra-arterial therapy may be considered for downstaging in selected patients with bilobar NELM. Adjuvant liver-directed therapy for residual post-cytoreduction disease is used in practice but has no prospective evidence base.

**Figure 2:**
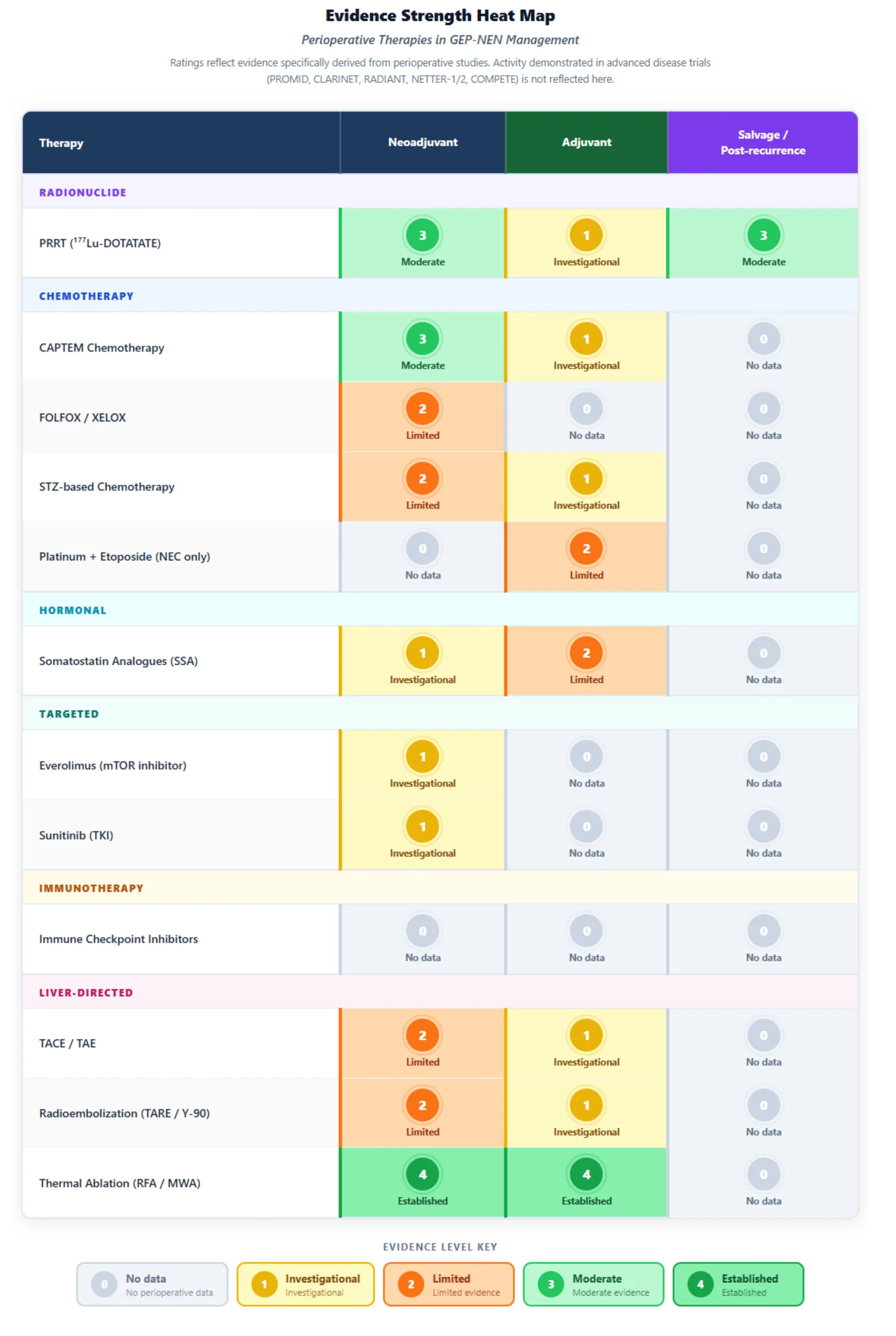
Evidence strength heat map. Notes: Evidence ratings reflect data specifically from perioperative studies. Thermal ablation is rated “Established” in the neoadjuvant/intraoperative column as a complementary intraoperative adjunct to hepatic resection. Platinum + etoposide adjuvant rating applies exclusively to poorly differentiated NEC. “Salvage/Post-recurrence” column reflects evidence for therapy given at confirmed post-resection disease recurrence. Abbreviations: PRRT, peptide receptor radionuclide therapy; CAPTEM, capecitabine/temozolomide; STZ, streptozotocin; SSA, somatostatin analog; TKI, tyrosine kinase inhibitor; TACE, transarterial chemoembolization; TAE, transarterial embolization; TARE, transarterial radioembolization; RFA, radiofrequency ablation; MWA, microwave ablation; NEC, neuroendocrine carcinoma.

The relative strength of perioperative evidence across all therapeutic modalities discussed in this review is summarized in Figure 2.

**Table 1:** Evidence summary for neoadjuvant therapy in GEP-NENs.

Study	Design	n	Grade/Differentiation	Disease Setting	Therapy	ORR (%)	DCR (%)	R0/Conversion Rate	Follow-Up	Post-Op Morbidity	Key Conclusion
Yan et al. 2024 [45]	Systematic review and meta-analysis	NR (pooled)	Mixed G1–G3	Advanced/locally advanced GEP-NEN	PRRT, chemo, TKI	42%	—	60% (vs. 63% upfront; *p* = NS)	NR	Pooled—acceptable across studies	Safe; effective for downstaging but no significant R0 improvement
Lee and Kim 2025 [102]	Meta-analysis	NR (pooled)	Well-differentiated GEP-NET	Locally advanced/borderline resectable	PRRT	39.1%	89%	69%	NR	NR	Supports neoadjuvant PRRT for downstaging; favorable disease control
Partelli et al. 2024 [101] (NEOLUPANET)	Phase II prospective multicenter	31 enrolled; 29 resected	G1–G2 NF-pNET	Resectable/borderline resectable pNET	^177^Lu-DOTATATE	58% PR; 0% PD	100%	83% (24/29)	NR	72% any complication; 24% severe (Clavien–Dindo ≥ III); 0% perioperative mortality	Safe; lower POPF vs. historical upfront resection; supports prospective evaluation
Partelli et al. 2018 [103]	Retrospective cohort	NR	G1–G2 pNEN	Resectable/potentially resectable pNEN	PRRT	—	—	65%	NR	POPF: 25% vs. 65% upfront (*p* = 0.011); overall morbidity comparable	Safe; significantly lower POPF rate vs. upfront resection
Parghane et al. 2021 [119]	Prospective cohort	57	Mixed G1–G3 GEP-NET	Locally advanced/unresectable (vascular involvement)	^177^Lu-DOTATATE	40% converted to resectable	—	40% primary tumors converted to resectable	2 yr OS 92.1%	Acceptable; no perioperative mortalities reported	Moderate conversion rate; tumor size, LN status, FDG uptake predict conversion
van Vliet et al. 2015 [120]	Retrospective cohort	29	G1–G2 NF-pNET	Initially unresectable pNET	^177^Lu-octreotate	—	—	Selected patients converted	NR	Acceptable; comparable to upfront resection	Valuable option for initially unresectable NF-pNET; supports conversion strategy
Squires et al. 2020 [67]	Retrospective cohort	NR	G1–G2 pNET	Locally advanced/resectable metastatic pNET	CAPTEM	43% PR	97%	87% proceeded to resection	Median PFS 28.2 mo; 5 yr OS 63%	Acceptable; no excess perioperative complications reported	Useful in borderline resectable or metastatic pNET; supports CAPTEM neoadjuvant role
Ambe et al. 2017 [66]	Retrospective cohort (single center)	NR	G1–G2 pNET	Borderline resectable pNET	CAPTEM ± RT	67% PR	100%	67%	NR	Acceptable; no excess surgical complications	Feasible in borderline resectable pNETs; supports multimodality approach
Maratta et al. 2025 [68]	Retrospective cohort	32	G1–G2 WD GEP-NET	Advanced (high tumor burden); 28% downstaged to resectability	FOLFOX/XELOX + SSA	—	87.5%	28% downstaged to radiographic resectability	NR	NR	Promising disease control; notable downstaging rate supports neoadjuvant role for oxaliplatin regimens
Prakash et al. 2017 [59]	Retrospective cohort	NR	G1–G2 pNET	Locoregionally advanced pNET	STZ/5-FU/Doxorubicin	7% PR	97%	64%	NR	NR	Primarily disease stabilization; modest objective response; still achieved surgical resection in majority

**Table 2:** Evidence summary for adjuvant therapy in GEP-NENs.

Study	Design	n	Grade/Differentiation	Disease Setting	Adjuvant Therapy	RFS/DFS vs. No Therapy	OS vs. No Therapy	Follow-Up	Key Conclusion
Barrett et al. 2020 [47]	Retrospective multicenter cohort	1871 (91 received adjuvant therapy)	Mixed G1–G3 GEP-NET	Curative-intent resection	SSA or cytotoxic chemotherapy	Worse RFS (5 yr RFS 36% vs. 81%)	No difference	Median NR	No benefit; chemotherapy associated with inferior RFS
Guo et al. 2024 [77]	Retrospective multicenter cohort	NR	G2 pNET (high recurrence risk)	Radical resection; no metastatic disease	Long-acting octreotide	No difference overall; possible benefit in LN+ or Ki-67 < 10% subgroup	No difference	NR	No benefit in overall cohort; subgroup findings hypothesis-generating only
Gao et al. 2020 [76]	Retrospective cohort	130	G2 pNET	R0/R1 radical resection	Long-acting octreotide	Improved RFS (24-mo DFS 98.3% vs. 88.7%; *p* = 0.037)	Not reported	NR	Potential RFS benefit; limited by retrospective design and absence of SSTR data
Wang et al. 2021 [78]	Retrospective cohort	NR	pNET (G3, PD dilation, or perineural invasion subgroup)	Resected pNET (high-risk pathological features)	Adjuvant SSA	Lower recurrence rate in high-risk subgroup (*p* < 0.001)	Not reported	NR	Hypothesis-generating; potential benefit in high-risk pathological subgroups only
Maire et al. 2009 [70]	Retrospective cohort	NR	WD GEP-NET	Post-hepatic resection for liver metastases	STZ/5-FU	No difference (5 yr RFS 20% vs. 38%; *p* = 0.36)	No difference (5 yr OS 96% vs. 76%; *p* = 0.58)	5 years	No benefit from adjuvant chemotherapy after hepatic resection
Xie et al. 2020 [48]	Retrospective multicenter cohort	NR	WD pNET (G1–G3)	Curative-intent resection of localized pNET	All perioperative systemic therapies (incl. targeted agents)	No difference	No difference	NR	No OS benefit from any perioperative systemic therapy modality in localized pNET
Merola et al. 2020 [121]	Retrospective multicenter cohort	NR	G3 NET/NEC	Radical surgery for G3 NEN	Adjuvant chemotherapy	No difference in G3 NET	No significant difference in NEC (40 vs. 19 mo; *p* = 0.35)	NR	No benefit in G3 NET; numerically improved but nonsignificant OS in NEC subgroup

Studies evaluating treatment given after curative-intent resection in patients with no clinical evidence of residual disease.

**Table 3:** Evidence summary for salvage therapy following post-resection.

Study	Design	n	Grade/Differentiation	Disease Setting	Salvage Therapy	PFS vs. Comparator	OS vs. Comparator	Follow-Up	Key Conclusion
Borbon et al. 2025 [104]	Retrospective cohort	NR	Mixed GEP-NET (G1–G3)	Recurrent or progressive disease after prior GEP-NET resection	PRRT (^177^Lu-DOTATATE)	Improved PFS (32.4 vs. 11.0 mo; *p* < 0.001)	Improved OS (49.8 vs. 38.4 mo; *p* = 0.009)	NR	PRRT improves survival in post-resection recurrence; supports role as salvage therapy
Kiritani et al. 2020 [122]	Retrospective cohort	44 (28 with recurrence)	Mixed WD-NET	Recurrent NELM after prior curative hepatectomy	Repeat hepatectomy vs. No repeat resection	Not specifically reported	Improved OS with repeat hepatectomy (HR 5.0 for death without repeat hepatectomy *p* = 0.036)	NR	Repeat hepatectomy is feasible and independently associated with improved survival in selected patients with recurrent NELM

Studies evaluating treatment initiated at confirmed disease recurrence or progression following prior GEP-NEN resection. These are distinct from adjuvant studies and should not be interpreted as evidence for postoperative therapy. Abbreviations: ORR, objective response rate; DCR, disease control rate; RFS, recurrence-free survival; DFS, disease-free survival; OS, overall survival; PFS, progression-free survival; PRRT, peptide receptor radionuclide therapy; NF-pNET, nonfunctioning pancreatic neuroendocrine tumor; WD, well-differentiated; GEP-NEN, gastroenteropancreatic neuroendocrine neoplasm; GEP-NET, gastroenteropancreatic neuroendocrine tumor (well-differentiated); CAPTEM, capecitabine/temozolomide; STZ, streptozotocin; 5-FU, fluorouracil; RT, radiation therapy; SSA, somatostatin analog; FOLFOX, folinic acid/fluorouracil/oxaliplatin; XELOX, capecitabine/oxaliplatin; POPF, postoperative pancreatic fistula; NELM, neuroendocrine liver metastases; NEC, neuroendocrine carcinoma; NET, neuroendocrine tumor; LN, lymph node; SSTR, somatostatin receptor; NR, not reported; NS, not significant; PR, partial response; PD, progressive disease; HR, hazard ratio.

#### 5.6.4. Surgery and Adjunct Therapy in NET G3 and NEC

As mentioned, the role of surgical resection in G3 NETs and NECs remains controversial, with retrospective studies demonstrating variable survival benefits depending on tumor differentiation and the use of adjuvant therapy. A multicenter retrospective study of patients with G3 NETs who underwent radical surgery found no benefit of adjuvant chemotherapy in terms of OS or RFS [121]. However, 2-year OS rates were significantly higher in patients with G3 NETs compared to those with NEC (75.6% vs. 53.1%), suggesting that surgery may offer a greater benefit in well-differentiated tumors [121]. Although a numerical difference in median OS was observed in the NEC subgroup receiving adjuvant therapy (40 vs. 19 months), this was not statistically significant (*p* = 0.35), raising the possibility that systemic therapy, rather than surgery alone, may drive outcomes in poorly differentiated disease [121].

## 6. Ongoing Trials and Future Directions

**Figure 3:**
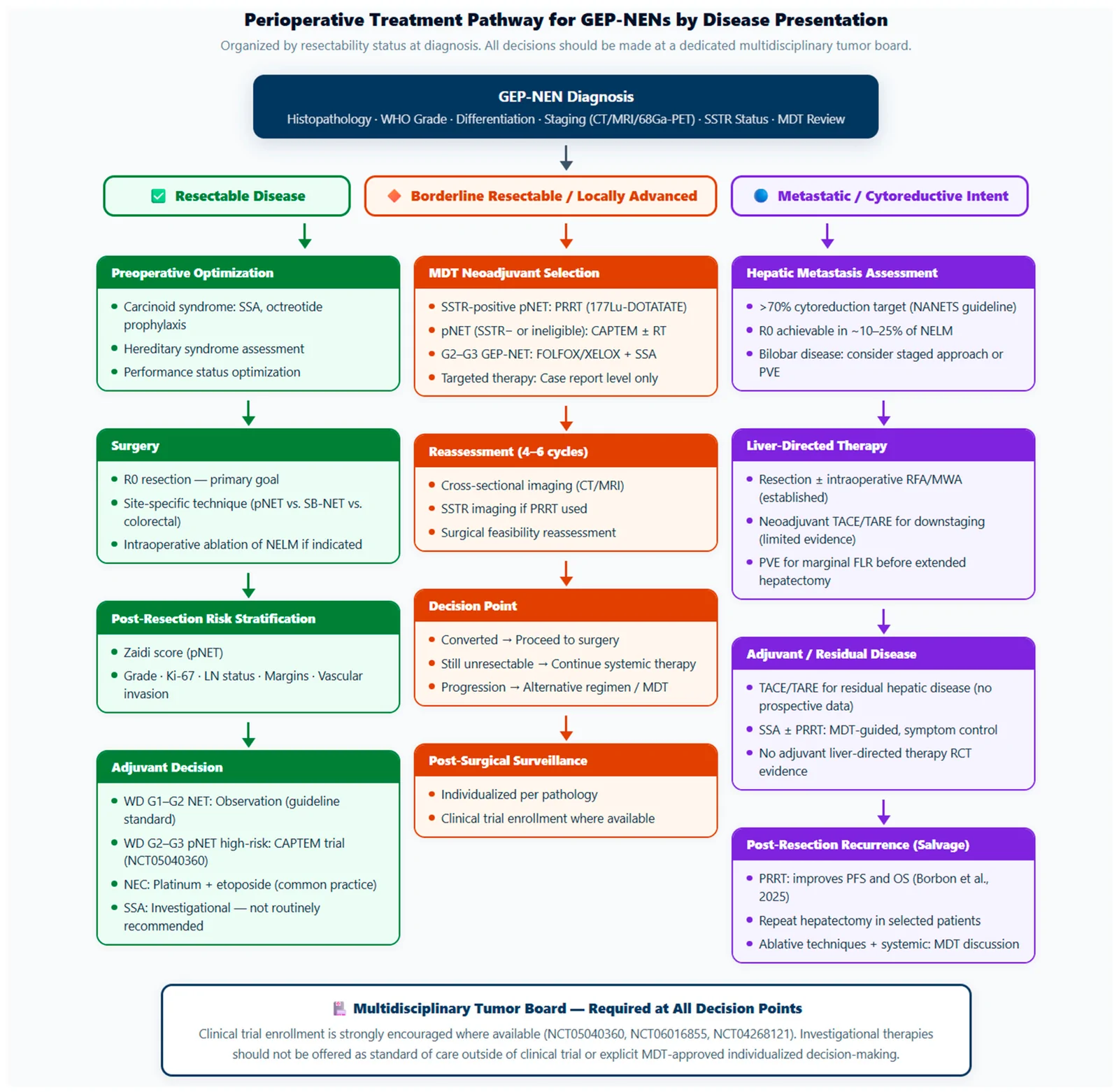
Perioperative treatment pathway for GEP-NENs by disease presentation. Abbreviations: GEP-NEN, gastroenteropancreatic neuroendocrine neoplasm; SSTR, somatostatin receptor; MDT, multidisciplinary team; PRRT, peptide receptor radionuclide therapy; CAPTEM, capecitabine/temozolomide; RT, radiation therapy; FOLFOX, folinic acid/fluorouracil/oxaliplatin; SSA, somatostatin analog; NEC, neuroendocrine carcinoma; NELM, neuroendocrine liver metastases; TACE, transarterial chemoembolization; TARE, transarterial radioembolization; PVE, portal vein embolization; FLR, future liver remnant; RFA, radiofrequency ablation; MWA, microwave ablation; pNET, pancreatic neuroendocrine tumor; SB-NET, small bowel neuroendocrine tumor; LN, lymph node; WD, well-differentiated; CT, computed tomography; MRI, magnetic resonance imaging; PET, positron emission tomography [104].

While a proposed perioperative treatment pathway based on current evidence is illustrated in Figure 3, there are several ongoing clinical trials looking at surgery and adjuvant therapies in GEP-NETs. NCT06016855 is an actively recruiting phase IV trial evaluating the efficacy of PRRT after surgical debulking in patients with metastatic GEP-NETs. Of particular importance in the adjuvant setting is the SWOG S2104 trial (NCT05040360), a phase II randomized controlled trial in high-risk pNETs comparing adjuvant CAPTEM to observation. This trial represents the first prospective randomized evaluation of adjuvant cytotoxic chemotherapy specifically in resected pNETs and remains actively recruiting. The COMPETE trial (NCT03049189) has recently reported positive results demonstrating superiority of ^177^Lu-edotreotide over everolimus for PFS in progressive G1/G2 GEP-NETs with ongoing regulatory review [100]. The COMPOSE trial (NCT04919226) is a phase III study evaluating ^177^Lu-edotreotide in patients with well-differentiated, aggressive G2 or G3 SSTR-positive GEP-NETs—a higher-grade population than COMPETE—and is currently ongoing [123]. Finally, the NEONEC trial is a phase II study assessing the efficacy of 12-month neoadjuvant chemotherapy in patients with locally advanced digestive NEC with relapse-free survival as the primary endpoint (NCT04268121).

## 7. Conclusions

The role of adjunct therapies in GEP-NENs is increasingly relevant as both systemic and liver-directed modalities continue to evolve. The investigation of these additional therapies in a surgical context is paramount, as surgery remains the backbone of treatment. The evidence, however, remains primarily retrospective and heterogeneous between modalities and endpoints, making direct comparisons between treatment options in the context of surgery a challenge. Neoadjuvant therapy shows the most promise in selected patients with borderline resectable or locally advanced GEP-NETs, with the strongest evidence supporting PRRT in SSTR-positive pNETs and CAPTEM-based chemotherapy for pancreatic primaries. However, the evidence base across modalities remains largely retrospective, and the consistent finding that neoadjuvant therapy does not reliably improve R0 resection rates tempers conclusions about meaningful surgical impact. Adjuvant systemic therapy lacks high-quality evidence of benefit in well-differentiated GEP-NETs and is not routinely endorsed by major guidelines, except for platinum-based chemotherapy following resection of NEC. Liver-directed therapies play an important complementary role in selected patients with hepatic metastases but have no prospective evidence in the formal adjuvant setting. Ultimately, the strength of available evidence varies considerably across therapeutic modalities and clinical settings. Neoadjuvant PRRT and CAPTEM have the most robust perioperative evidence, while adjuvant systemic therapy, targeted agents, and immune checkpoint inhibitors remain investigational in the perioperative context. Optimal management of GEP-NENs requires multidisciplinary evaluation, treatment plans individualized to tumor biology, differentiation, primary site, and patient goals, and prioritization of clinical enrollment to generate the prospective data currently needed.

## Data Availability

The original contributions presented in this study are included in the article. Further inquiries can be directed to the corresponding author.

## Abbreviations

The following abbreviations are used in this manuscript:

GEP-NET	Gastroenteropancreatic neuroendocrine tumor
pNET	Pancreatic neuroendocrine tumor
PRRT	Peptide receptor radionuclide therapy
SSA	Somatostatin analogues
NEC	Neuroendocrine carcinoma
